# Protective effect of epigallocatechin‐3‐gallate against neuroinflammation and anxiety‐like behavior in a rat model of myocardial infarction

**DOI:** 10.1002/brb3.1633

**Published:** 2020-04-18

**Authors:** Jinpeng Wang, Ping Li, Tian Qin, Dongjie Sun, Xin Zhao, Beilin Zhang

**Affiliations:** ^1^ Department of Cardiology The Second Hospital of Jilin University Changchun China; ^2^ Department of Paediatrics The Second Hospital of Jilin University Changchun China; ^3^ Department of Physiology College of Basic Medical Sciences Jilin University Changchun China

**Keywords:** antianxiety effect, EGCG, hippocampus, myocardial infarction rat, neuroinflammation

## Abstract

**Objective:**

Individuals who experience myocardial infarction (MI) often experience anxiety. Green tea has potent antioxidative properties and, epigallocatechin‐3‐gallate (EGCG), which is a primary component of tea polyphenols, has advantageous effects on anxiety and depression. However, its mechanism of action regarding the inhibition of anxiety‐like symptoms after MI remains unclear. This study examined whether EGCG alleviated anxiety‐like behavior in MI rats and its possible mechanism.

**Material and Methods:**

Rats were administered a daily gavage of EGCG (50 mg/kg) 7 days before and 14 consecutive days after the MI procedure. The open‐field test and light/dark shuttle box were performed to evaluate anxiety‐like behavior. Serum and hippocampus interleukin (IL)‐6 levels were tested using ELISA. Caspase 3, caspase 8, caspase 9 and bcl‐2 messenger RNA levels in the hippocampus were determined using quantitative polymerase chain reaction, and STAT3 protein was detected by Western blot.

**Results:**

Results of the open field test and light/dark shuttle box task demonstrated that the MI procedure induced anxiety‐like behavior in the animals, and this impairment was improved by EGCG. Daily EGCG administration significantly decreased the level of IL‐6 both in serum and hippocampus after MI. The administration of EGCG also significantly moderated the expression of caspases 3, 8, and 9 mRNA, which was related to apoptosis in the hippocampus. Furthermore, EGCG also downregulated the expression of STAT3, which was related to the activity of IL‐6. These results suggest that EGCG alleviated anxiety‐like behavior by inhibiting increases in neuroinflammation and apoptosis in the rat hippocampus. In addition, EGCG reversed alterations of IL‐6 and STAT3 in the brain to alleviate apoptosis in the hippocampus.

**Conclusions:**

Thus, EGCG reversed anxiety‐like behavior through an anti‐inflammation effect to alleviate apoptosis in neurons and may be a useful therapeutic material for anxiety‐like behavior after MI.

## Graphical abstract

Epigallocatechin‐3‐gallate reversed anxiety‐like behavior in myocardial infarction (MI) rats through an anti‐inflammation effect by alleviating neuronal apoptosis and may be a useful therapeutic agent for anxiety‐like behavior after MI.

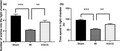

## 1 INTRODUCTION

Individuals who experience myocardial infarction (MI) also often experience anxiety (Havik & Maeland, 1990; Lane, Carroll, Ring, Beevers, & Lip, 2002) and depression (Thombs et al., 2006). Akhtar, Malik, & Ahmed (2004) reported that up to 50% patients who experience acute MI have been found to experience symptoms of anxiety and/or depression 1 week later. It has often been reported that depression after MI is associated with adverse outcomes (Meijer et al., 2013); however, the contribution of anxiety to this process after MI has not been extensively investigated. A recent meta‐analysis found that patients who experienced anxiety symptoms after MI had a 71% increased risk for a new cardiovascular event, a 47% increased risk for death, and a 36% increased risk for a new cardiovascular event or death (Roest, Martens, Denollet, & Jonge, 2010). Therefore, post‐MI anxiety is an important risk factor for new cardiovascular events or death.

Nevertheless, the mechanism of anxiety after MI remains unclear, although studies have reported that MI is closely related to inflammation. After MI, immune‐induced inflammation, including complement activation, cytokine release, inflammation and immune cell chemotaxis and infiltration, can aggravate myocardial injury and expand the scope of MI. Other studies have shown that the use of anti‐inflammatory drugs, such as pentoxifylline, can reduce apoptosis in the limbic system of the brain after MI (Bah, Kaloustian, Rousseau, & Godbout, 2011). At the same time, not only myocardial apoptosis, but also apoptosis in neurons of the hippocampus and amygdala increased after MI (Wann et al., 2009). As a part of the limbic system, the hippocampus is involved in the pathophysiology of affective disorders, fear, and anxiety behavior (Cho, Rhee, Kwack, Chung, & Kim, 2008; Kjelstrup et al., 2002; McHugh, Deacon, Rawlins, & Bannerman, 2004; Wang, Li, & Zhu, 2007). It has been reported that reduction in anxiety‐like behavior induced by 3,4‐methylenedioxy‐methamphetamine (MDMA) can control hippocampus apoptosis (Karimi, Jahanshahi, & Golalipour, 2014). This indicates that apoptosis in the hippocampus may have a relationship with anxiety‐like behavior. Therefore, anxiety‐like behavior after MI may be related to inflammation‐induced apoptosis in hippocampal neurons.

Epigallocatechin‐3‐gallate (EGCG) is one of the main components of tea polyphenols in green tea, which have prospects for broad application in medicine and biology. Polyphenols have functions of regulating immunity, anti‐inflammation, antitumor, antiatherosclerosis, and antioxidation (Frei & Higdon, 2003; Fuss et al., 2004; Miura et al., 2001; Tedeschi et al., 2004; Yang et al., 2001). EGCG has been demonstrated to exhibit neuroprotective functions through attenuation of inflammation in cerebral ischemia/reperfusion injury. Furthermore, studies have shown that EGCG can reduce the area of myocardial injury after ischemia‐reperfusion (I/R) in diabetic rats (Wu et al., 2017). Xuan et al. showed that EGCG has protective effects on myocardial I/R injury in rats. This may be due to the inhibition of apoptosis by EGCG through several targets of the PI3K/Akt signaling pathway and the restoration of autophagic flux to alleviate myocardial I/R injury (Xuan & Jian, 2016). More importantly, EGCG has neuroprotective effects on rat hippocampal radiation injury and apoptosis (El‐Missiry, Othman, El‐Sawy, & Lebede, 2018). Therefore, EGCG may have the neuroprotective effect by attenuation of inflammation after MI.

Therefore, the aim of the present study was to investigate whether EGCG could mitigate anxiety‐like behavior in a rat model of MI and to potentially identify its mechanism of action. It was hypothesized that the anti‐inflammation effect of EGCG may suppress hippocampal neuron apoptosis which may contribute to its antianxiety effects.

## 2 MATERIAL AND METHOD

### 2.1 Animal models

Thirty‐six male Wistar rats were acquired from a commercial supplier (Changchun Yisi Laboratory Animal Technology Co., Ltd) and randomly divided into 3 groups: sham, MI, and EGCG. All experimental rats were fed in the cages with water and food ad libitum under the conditions of appropriate temperature (23 ± 1°C), humidity (55 ± 5%), and 12‐hr light/dark alternating. The protocols were carried out in consonance with the Institutional Guidelines for Care and Use of Laboratory Animals of NIH Publication (No. 85‐23, revised 1996). The Ethics Committee of Medical Experiment Animals in the College of Basic Medicine of Jilin University (Changchun, China) endorsed all animal assays in this research (No. 2019070).

Rats were ligated around the left anterior descending coronary artery on the basis of the methods from earlier research (Yu, Yin, Bao, & Guo, 2018) to establish MI model group. Rats in EGCG group also underwent ligation of the left ventricular coronary artery. Rats in the sham group underwent thoracotomy and received same treatment without performing coronary artery ligation. All rats were administered with 1.6 × 107 units of penicillin (intraperitoneal) immediately after surgery and 0.4 × 107 units of penicillin was utilized for additional 2 days following the MI procedure.

Rats in the sham and MI groups were administered with a normal saline as vehicle (0.9% NaCl) by daily gavage. Rats in the EGCG group were administered with 50 mg/kg EGCG (Shanghai Yuan Ye Biotechnology Co., Ltd.). All administrations began 7 days before and were continued for 14 days after the procedure.

For the evaluation of the MI injury, the experimental rats were connected to a multichannel biological signal analysis system (Powerlab, AD Instruments) to collect electrocardiogram data from the sham and MI groups. ST‐segment elevation was considered to be the successful establishment of MI animal model.

### 2.2 Behavioral tests

#### 2.2.1 Light–dark shuttle box

The light–dark shuttle box test is applied to reflect the combination of danger and risk aversion (Barr & Forster, 2011), which is a typical laboratory device for assessing an anxiety‐like behavior. The equipment was made up of a laboratorial box and automatic recording and printing device. The laboratorial box is a white‐black rectangular plexiglass box (length × width × height: 46 × 27 × 30 cm), which is separated into two compartments via a baffle (light compartment: 27 × 27 cm, dark compartment: 18 × 27 cm). A 7.5 × 7.5 cm opening was made at the bottom of the partition to separate the light and dark compartments. First, we performed the light–dark shuttle box test for 2 days. Then, we performed the open field test (OFT). The OFT was performed for 2 days. The light–dark shuttle box test was implemented relying on a previously published protocol (Bourin & Hascoet, 2003). Each experimental rat was placed in the center of the light compartment and faced away from the opening. The rats were allowed to explore the box for 5 min; meanwhile, the time spending in the light compartment and the entered frequency into the light compartment of these rats were automatically recorded by utilizing a computer‐controlled detection equipment. The experimental equipment was cleaned after each trial.

#### 2.2.2 Open field test

OFT was carried out to evaluate the locomotor, exploratory, and anxiety‐like behaviors in rodents (Lane et al., 2002). The experimental rats were placed in the area of the open‐field squares (100 × 100 × 40 cm). The open‐field squares were averagely divided into 9 squares, which were composed of gray nonreflecting base and walls. The central square was defined as the “center” area. Every experimental rat was placed in the corner of the open‐field square, as well as observed and recorded the total travelled distance, total times of the animal entered the center zones, and the average velocity of the animal during 5 min. At the end of each trial, the experimental equipment was cleaned by utilizing 70% alcohol and dried to remove the scents from other rats.

### 2.3 Blood and tissue sample collection

After the behavior test, all rats were decapitated between 12:00 and 13:00, and the blood from the trunks was collected. Simultaneously, the hippocampus was rapidly separated. The serum and hippocampus were stored at −80°C until analyzed.

### 2.4 Estimation of IL‐6 in the brain and blood by ELISA

The levels of interleukin‐6 (IL‐6) in serum and hippocampus were determined using commercially available rat ELISA assay kits (R&D SYSTEM), relaying on the product description.

### 2.5 Real‐time fluorescent quantitative polymerase chain reaction for caspases 3, 8, 9, and bcl‐2 in the hippocampus

Conforming to the kit specification, total RNA was isolated from hippocampus using a commercially available kit (TRIzol reagent, Invitrogen). RNA with purity A260/280 ≥1.8 without the contamination of genomic DNA was resolved on 1% agarose gel to assess the integrity of 5S, 18S, and 28S ribosomal RNA through adopting a spectrophotometer (Eppendorf PhysioCare Concept). The concentration of RNA was adjusted for compounding the complementary DNA (cDNA) (Vazyme Biotech Co., Ltd). The targeted genes and primers are summarized in Table 1. The thermal cycle program consisted of 3 s at 95°C, 40 cycles at 10 s at 95°C, and 1 min at 60°C. Expression levels of target genes were normalized to the expression of GAPDH mRNA.

**TABLE 1:** Gene primer sequences and product size used for GAPDH, caspase 3, caspase 8, caspase 9, and Bcl‐2

Gene	Oligonucleotides used for qPCR primer sequence 5′‐3′
*Caspase 3*	F: TGGACAACAACGAAACCTC
	R: ACACAAGCCCATTTCAGG
*Caspase 8*	F: CACATCCCGCAGAAGAAG
	R: GATCCCGCCGACTGATA
*Caspase 9*	F: GGAGTTGACTGAGGTGGGA
	R: AGCAAGGAAGACACTGGGA
*Bcl‐2*	F: CGGGAGAACAGGGTATGA
	R: AGGCTGGAAGGAGAAGATG
*GAPDH*	F: GACATGCCGCCTGGAGAAAC
	R: AGCCCAGGATGCCCTTTAGT

Abbreviations: F, forward; GAPDH, glyceraldehyde 3‐phosphate dehydrogenase; qPCR, quantitative polymerase chain reaction; R, reverse.

### 2.6 Western blot analysis

Hippocampal tissues were incubated in 1 ml RIPA lysis buffer for 30 min to extract total protein. The 20 μg protein lysate was applied for conducting sodium dodecyl sulfate–polyacrylamide gel electrophoresis and then was proceeded the polyvinylidene difluoride membranes transferring. After sealing for 1 hr in the mixed solution of phosphate‐buffered solution (PBS), Tween‐20, and 5% nonfat milk, the membranes were incubated with the related primary antibody against STAT3 (Cell Signaling Technology 1:2,000) at 4°C overnight. After 3 rinses with PBS, the proper secondary antibodies of Peroxidase‐Conjugated Goat Anti‐Mouse IgG (H + L) (1:15,000 dilution, YEASEN, cat: 33201ES60) and ProteinFindTM Goat Anti‐Rabbit IgG (Transgen, cat: HS101‐01) were subsequently utilized to culture with above membranes for 60 min at room temperature following by 3 rinses with PBS. Protein was quantified using a scanning laser densitometer (Biomed Instruments Inc.), and the gray value of the bands was simultaneously analyzed. Protein level of STAT3 was expressed as arbitrary units after normalization with GAPDH protein expression.

### 2.7 Statistical analysis

The data were expressed as the mean ± standard error of the mean. One‐way analysis of variance was used for the statistical evaluations. Significant effects were assessed using Tukey's multiple test, with *p* < .05 being considered as statistically significant. Data were analyzed using SPSS 18.0 software (IBM SPSS Statistics).

## 3 RESULTS

### 3.1 Survival and identification of MI model

Six of 12 MI rats treated with saline died (50% mortality). Four of 15 MI rats treated with EGCG died. Only one sham rat died during experimentation. The MI and EGCG rats observed with characteristic MI alternations in the ECG pattern including elevation of the ST segment shown in Figure S1.

### 3.2 Light–dark shuttle box and OFT

**FIGURE 1:**
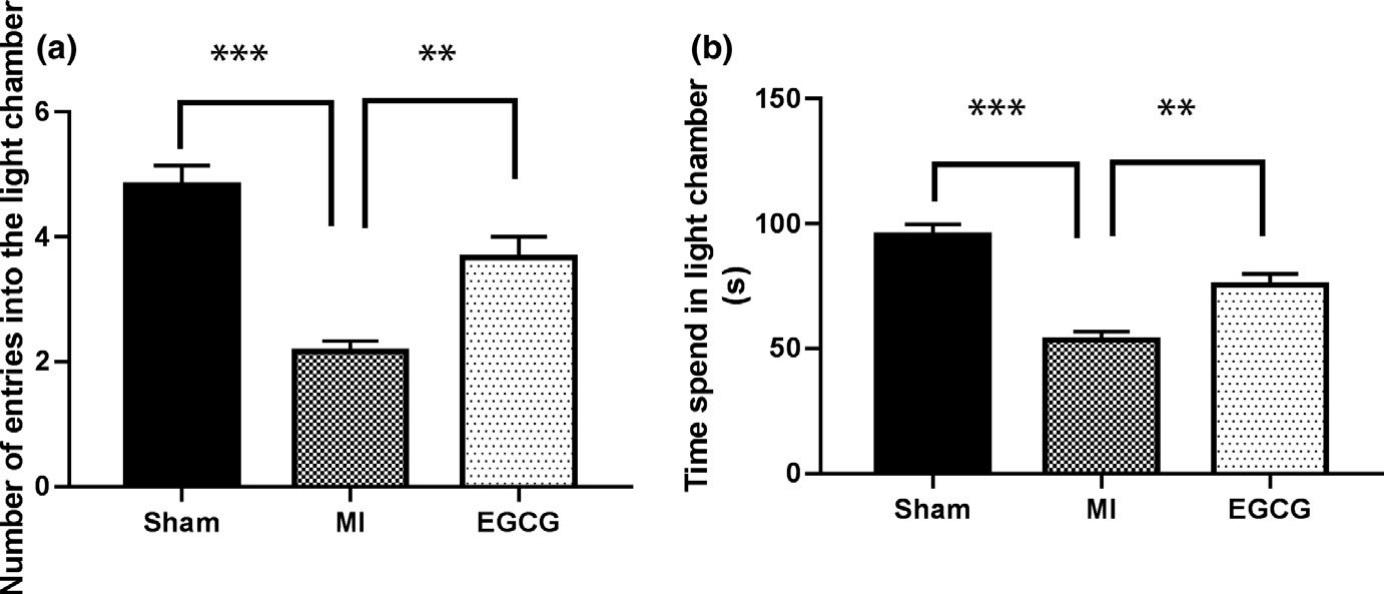
Reduced anxiety‐like behavior in rats with myocardial infarction (MI) administered epigallocatechin‐3‐gallate (EGCG) in the light–dark box test. The bar graphs show the total time (seconds) spent in the light area and total number of transitions during a 5‐min trial. (a) Number of entries into the light chamber. (b) Time spend in light chamber. Values are expressed as median ± standard error of the mean of 6 rats per group. **p < .01, and ***p < .001 indicate significant values compared with the MI group (one‐way analysis of variance with Tukey's multiple test)

Data from the light–dark shuttle box experiments are presented in Figure 1. The time spent in the light compartment by rats in the MI group (53.27 ± 3.54 s) was a significantly decreased compared with sham (95.53 ± 4.29 s; *p* < .001) and those in the EGCG (75.33 ± 4.66 s; *p* < .01) groups. The number of entries into light compartment (2.167 ± 0.167) by rats in the MI group was also decreased compared with sham‐ (4.833 ± 0.31 s; *p* < .001) and EGCG‐treated (3.667 ± 0.333 s; *p* < .01) animals (Figure 1).

**FIGURE 2:**
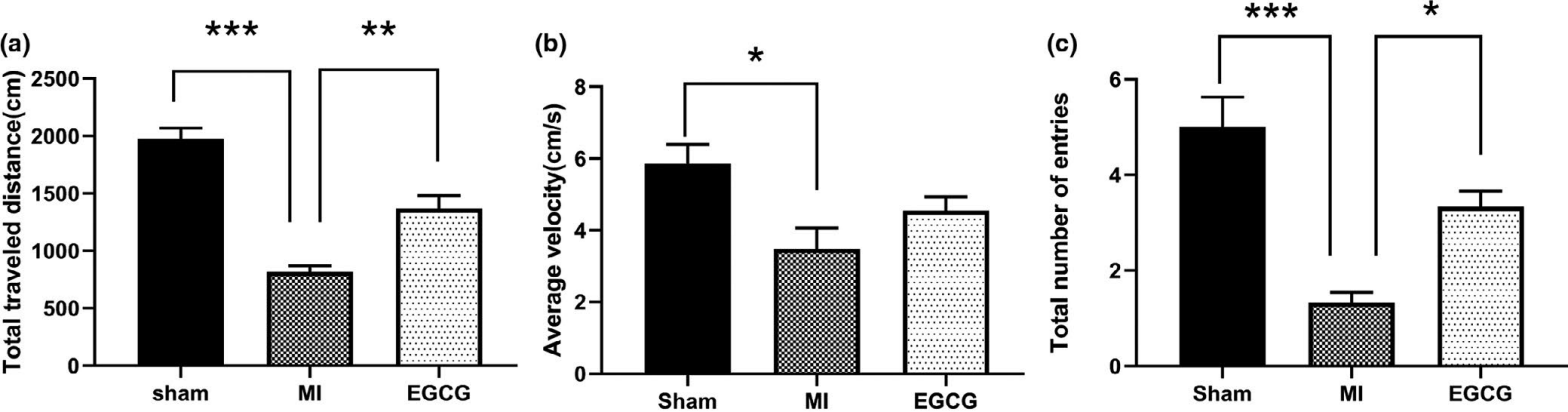
The effect of epigallocatechin‐3‐gallate (EGCG) administration on rats with myocardial infarction (MI) in the open‐field test. (a) Total travel distance was decreased in rats with MI while EGCG application reversed that behavior comparable with levels observed in MI rats without pharmacological intervention. (b) Average velocity. (c) The total number of entries into the center zones. Values are expressed as mean ± standard error of the mean. *p < .05, **p < .01, and ***p < 0.001indicate significant values compared with the MI group (one‐way analysis of variance with Tukey's multiple test)

The OFT is one of the most popular ethological tests to assess anxiety‐like behavior in rodents. The total traveled distance has been reported to reflect anxiety‐like behavior. In the present study, MI rats exhibited significantly lower distance traveled compared with the sham (1,976 ± 914.48 cm; *p* < .001) and EGCG (1,367 ± 114.3 cm; *p* < .01, Figure 2) groups. The total number of entries into the center zones by rats in the MI group (1.33 ± 0.21) was also less than sham (5 ± 0.633; *p* < .001) and EGCG group (3.33 ± 0.333; *p* < .05). There was a significant difference in average velocity between the sham and MI groups (5.861 ± 0.534 versus 3.476 ± 0.589, respectively; *p* < .05).

### 3.3 IL‐6 levels in serum and hippocampus

**FIGURE 3:**
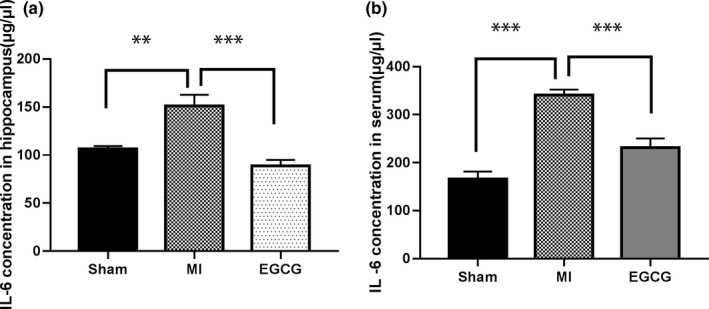
Epigallocatechin‐3‐gallate (EGCG) ameliorates inflammation and improves the IL‐6 in a rat model of myocardial infarction (MI). EGCG ameliorated IL‐6 levels in a rat model of MI. (a) Interleukin (IL)‐6 levels in the hippocampus. (b) IL‐6 levels in serum. Each column represents mean ± standard error of the mean for 6 rats in each group. **p < .01, and ***p < .001 indicate significant values compared with the MI group (one‐way analysis of variance with LSD test)

As shown in Figure 3a and b, the level of IL‐6 in the hippocampus was significantly increased in MI rats (151 ± 11.77 μg/μl) compared with sham (106.6 ± 2.917 μg/μl; *p* < .01). EGCG (88 ± 6.04) treatment reduced IL‐6 levels compared with MI rats (151 ± 11.77 μg/μl; *p* < .001). In the MI group, the serum levels of the proinflammatory cytokine IL‐6 (340.8 ± 11.31 μg/μl) significantly increased compared with the sham group (166 ± 15.45 μg/μl; *p* < .001). The EGCG group (231 ± 19.43 μg/μl) exhibited remarkably lower expression of IL‐6 in serum compared with the MI group (340.8 ± 11.31 μg/μl; *p* < .001).

### 3.4 Expression of caspases 3, 8, 9, and bcl‐2 mRNA in the hippocampus

**FIGURE 4:**
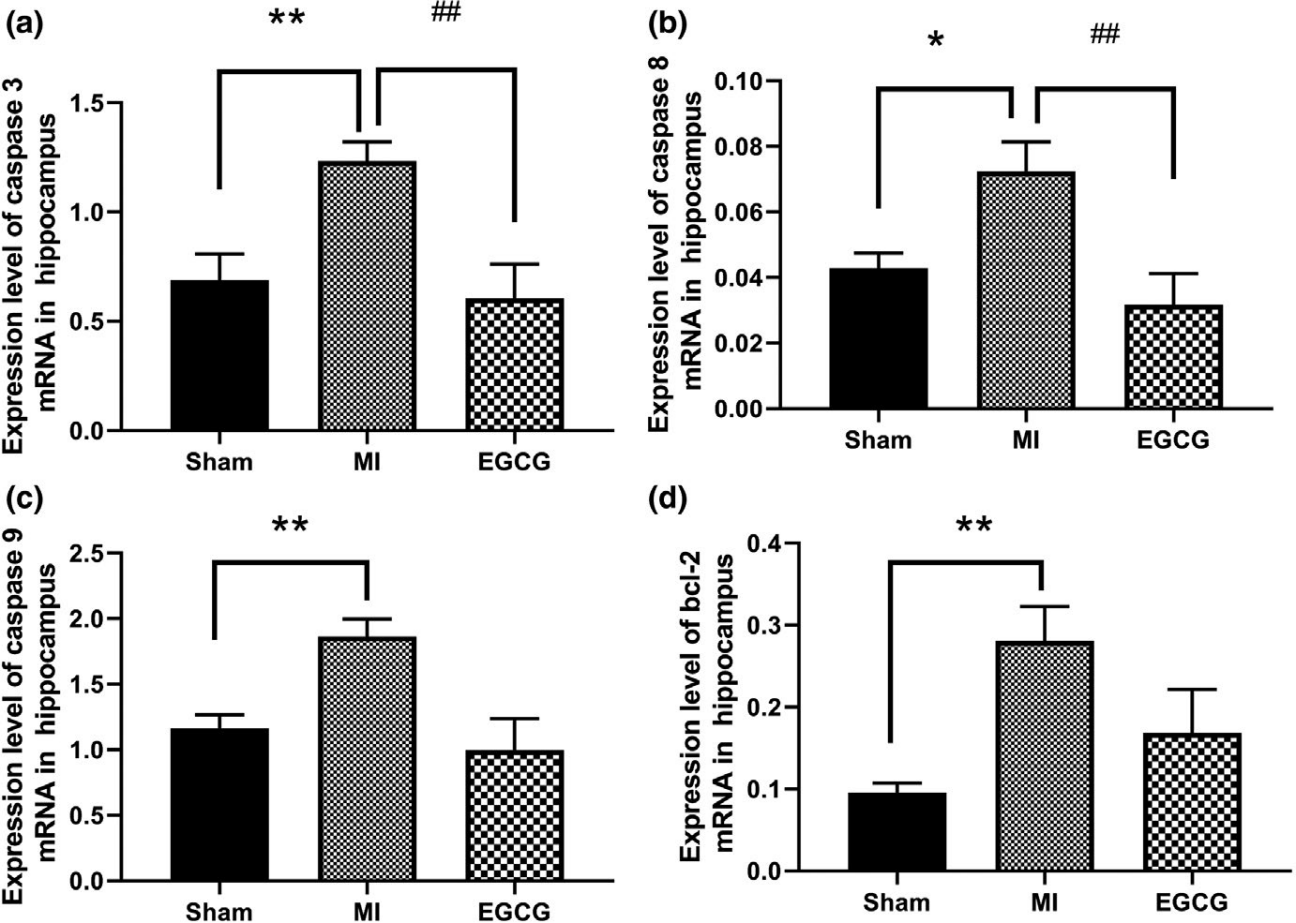
Messenger RNA levels of caspase 3, caspase 8, caspase 9, and bcl‐2 in the hippocampus of the sham, MI, and EGCG groups according to quantitative polymerase chain reaction. (a) Expression of caspase 3 mRNA; (b) Expression of caspase 8 mRNA; (c) Expression of caspase 9 mRNA; (d) Expression of bcl‐2 mRNA. Glyceraldehyde 3‐phosphate dehydrogenase (GAPDH) served as the internal control. Data presented as the mean ± *SEM*. *p < .05 versus MI group. **p < .01 Sham versus MI group. ##*p* < .01 MI versus EGCG group

mRNA expression of caspases 3, 8, and 9, and bcl‐2 in the hippocampus was analyzed. Caspase 3, caspase 8, caspase 9, and bcl‐2 mRNA expression were significantly upregulated in the MI group compared with the sham group (Figure 4). Additionally, EGCG downregulated caspase 3and caspase 8 mRNA expression compared with the MI group (Figure 4).

### 3.5 EGCG inhibited proinflammatory cytokine secretion through suppression of the STAT3 pathway

**FIGURE 5:**
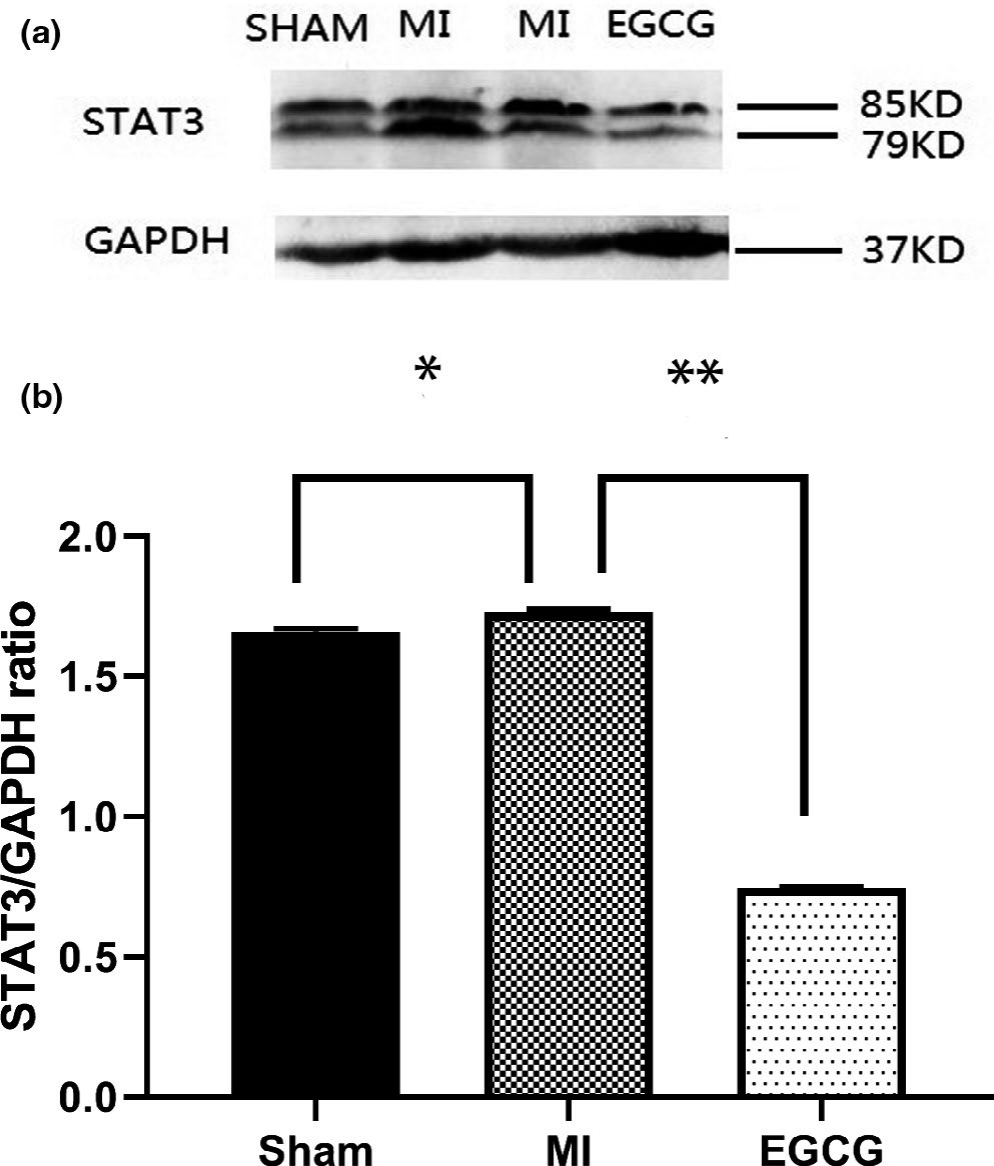
Western blot analysis of STAT3 activity in the hippocampus after myocardial infarction (MI). (a) Representative blots of each protein. (b) The relative abundance was obtained by normalizing the protein density against that of GADPH. Each column and bar represent mean ± *SEM*. Each point is an average of 4 separate experiments. *p < .05, **p < .01 compared with MI group

As shown in Figure 5, upregulation of STAT3 protein was confirmed in the MI group (1.73 ± 0.0104) compared with the sham group (1.66 ± 0.01, *p* < .01). When compared with EGCG‐treated rats, MI rats exhibited significantly higher STAT3 activation (EGCG: 0.7475 ± 0.004 vs. MI: 1.73 ± 0.0104, *p* < .001).

## 4 DISCUSSION

A previous prospective study reported that 41.0% exhibited anxiety symptoms, and 51% demonstrated both anxiety and depression among 288 patients with MI (Lane et al., 2002). Additionally, anxiety also had a negative correlation with the prognosis of post‐MI patients (Rafael, Simon, Drotos, & Balog, 2014). A related animal research revealed that the anxiety‐like behavior was increased in rats up to 4 weeks after MI; meanwhile, the interest in a new environment and the abilities of overall mobility and avoidance of social interaction were all reduced in rats after MI (Schoemaker & Smits, 1994). Our results from OFT assay demonstrated that rats in MI group had significantly lower travelled distance compared with the rats in sham and EGCG groups. Moreover, the total times of the rats entered the center zones in MI group were also less than that in sham and EGCG groups. Furthermore, evidence from the light–dark box assay disclosed that the time of the rats with MI in the light compartment was distinctly diminished as relative to the sham and EGCG groups. Likewise, the number of the MI rats entered into the light compartment was also abated compared with the sham and EGCG groups. Our results indicated that the rats exhibited anxiety‐like behavior after MI, which was alleviated by EGCG treatment.

The increase of anxiety‐like behavior after MI can be explained by neuronal apoptosis in hippocampal neurons. Different parts of the brain, especially the hippocampus, are involved in mediating anxiety (Cho et al., 2007, 2008; Wang et al., 2007). As a part of the limbic system, the hippocampus participates in the pathophysiological processes of emotional disorders, fear, and anxiety (Cho et al., 2007, 2008; McHugh et al., 2004; Wang et al., 2007). Evidence from Karimi et al. (2014) found that different doses of MDMA could cause different responses of anxiety‐like behavior, such as 2.5 mg/kg MDMA could control apoptosis in the hippocampus and at the same time reduce anxiety‐like behavior. This suggests that apoptosis in hippocampal neurons is associated with anxiety‐like behavior after MI. Apoptosis is one of the major pathways that can lead to the process of cell death after MI. Caspase family consists of a series of enzymes which are embroiled in apoptosis and/or inflammation. Furthermore, caspase family plays significant roles after MI, which may trigger the excessive apoptosis. Caspase 3 is an important cysteine protease, which is the effector molecule of DNA breakage and showcases a vital role to directly induce apoptosis. In a rat model of post‐MI syndrome, the important results uncovered that caspase 3 was both activated in the myocardium and limbic system (Boucher et al., 2004), which joined in modulating mood and emotions (Kaloustian et al., 2008; Wann et al., 2006). Caspases 8 and 9 have been corroborated to induce the activation of caspase 3, thereby regulating the course of cell apoptosis (Sugawara et al., 2004). Bcl‐2 family play a significant role in the intrinsic apoptosis pathway. Accordingly, we examined caspases 3, 8, 9, and bcl‐2 mRNA expressions in the hippocampus. Our results demonstrated that the levels of caspases 3, 8, 9, and bcl‐2 mRNA were all increased in the hippocampus after MI, which indicated the promotion of apoptosis genes in hippocampal neurons.

It has been certified that the crucial proinflammatory cytokines of TNF, IL‐6, and IL‐1β are consistently aggrandized after MI in the diverse animal models (Dewald et al., 2004; Herskowitz, Choi, Ansari, & Wesselingh, 1995). The upregulated IL‐ 6 is discovered in the infarcted myocardium activating the JAK/STAT cascade (Fischer & Hilfiker‐Kleiner, 2008). STAT3 is a momentous signaling pathway, which is observed to activate in traumatic brain injury, and moreover may partake in the mediation of the restoration of neurological function (Oliva, Kang, Sanchez‐Molano, Furones, & Atkins, 2012; Zhao, Zhang, Li, Su, & Hang, 2011). The impaired prohibition of IL‐6 receptor/STAT3 signaling has been testified to be linked to the prolonged and upgraded inflammation in a mouse model of MI, which augments the incidence of cardiac rupture (Hilfiker‐Kleiner et al., 2010). Our results showed that IL‐6 level was raised after MI, moreover, the expression of STAT3 protein was also increased after MI. Therefore, the upregulations of IL‐6 and STAT3 might be related to the pathogenesis of MI. Furthermore, one research disclosed the positively correlation between the neuronal apoptosis rate and the levels of TNF‐α and IL‐6 (Li et al., 2014). Other studies displayed that elevated levels of proinflammatory cytokines induced by post‐MI were accompanied by the occurrence of apoptosis in the hippocampus and amygdala (Kaloustian et al., 2009; Wann et al., 2006). Our results showed that apoptosis genes of hippocampal neurons in rats were associated with increased levels of IL‐6 by modulating STAT3 pathway after MI.

EGCG is known to be the overriding component of catechins, which emerges the multifarious protective functions depending on its antioxidative and antiapoptotic characters (Kaufmann, Henklein, Henklein, & Settmacher, 2009; Shimmyo, Kihara, Akaike, Niidome, & Sugimoto, 2008). Several studies have shown that EGCG inhibits the escalation of IL‐6 level in liver injury and exhibits a protective effect in cancer via the JAK/STAT3 pathway (Lee et al., 2004; Liu, Zhang, Jiang, Guo, & Zheng, 2014). Additionally, EGCG obviously restrained infrasound‐triggered microglial activation in the rat hippocampal region through diminishing IL‐1β, IL‐6, IL‐18, and TNF‐α expression and dampened infrasound‐evoked hippocampus neuronal apoptosis (Cai et al., 2014). Our results showed that MI rats with EGCG administration exhibited less anxiety‐like behavior compared with MI rats, and the levels of IL‐6 and STAT3 in the hippocampus were also abated in the EGCG group compared with that in the MI group. Beyond that, expression levels of caspases 3, 8, and 9 were likewise decreased in the EGCG group. Therefore, our research hinted that EGCG might suppress the level of IL‐6 by downregulating STAT3 pathway as well as inhibit apoptosis of hippocampal neuron by decreasing the level of IL‐6, thereby alleviating the anxiety‐like behavior after MI.

## 5 CONCLUSION

EGCG reversed anxiety‐like behavior in MI rats through an anti‐inflammation effect by alleviating neuronal apoptosis and may be a useful therapeutic agent for anxiety‐like behavior after MI.

## CONFLICT OF INTEREST

The authors declare that they have no conflict of interest.

## AUTHOR CONTRIBUTION

B.Z. and J.W. conceived and designed the experiments; T.Q., D.S., and X.Z. performed the experiments; B.Z. and J.W. analyzed the data; B.Z. and P.L wrote the paper; P.L and B.Z. involved in funding acquisitions.

## Supporting information

FIGURE S1

## Data Availability

The data that support the findings of this study are available from the corresponding author upon reasonable request.
