# Supplementary figures and images for: Protective effect of epigallocatechin‐3‐gallate against neuroinflammation and anxiety‐like behavior in a rat model of myocardial infarction
Source: Brain Behav. 2020 Apr 18;10(6):e01633. doi: 10.1002/brb3.1633 (PMC7303397; doi:10.1002/brb3.1633)

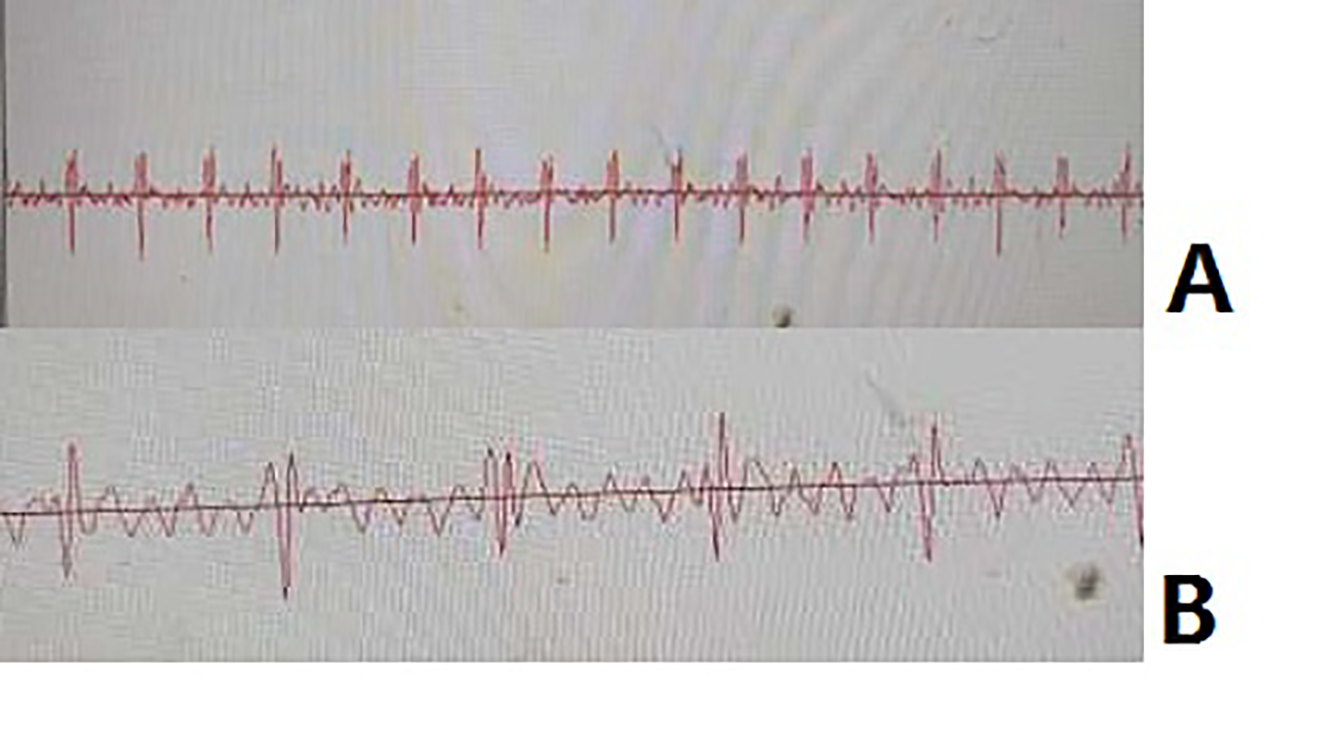

Supplement: Supplementary file 1 — FIGURE S1 [file BRB3-10-e01633-s001.tif]
